# A comparison of host gene expression signatures associated with infection *in vitro* by the Makona and Ecran (Mayinga) variants of Ebola virus

**DOI:** 10.1038/srep43144

**Published:** 2017-02-27

**Authors:** Andrew Bosworth, Stuart D. Dowall, Isabel Garcia-Dorival, Natasha Y. Rickett, Christine B. Bruce, David A. Matthews, Yongxiang Fang, Waleed Aljabr, John Kenny, Charlotte Nelson, Thomas R. Laws, E. Diane Williamson, James P. Stewart, Miles W. Carroll, Roger Hewson, Julian A. Hiscox

**Affiliations:** 1 National Infection Service, Public Health England, Porton Down, Salisbury, UK; 2 National Institute of Health Research, Health Protection Research Unit in Emerging and Zoonotic Infections, Liverpool, UK; 3 Institute of Infection and Global Health, University of Liverpool, UK; 4 High Containment Microbiology, Public Health England, Porton Down, Salisbury, UK; 5 School of Molecular and Cellular Medicine, University of Bristol, UK; 6 Centre for Genomics Research, University of Liverpool, UK; 7 Defence Science and Technology Laboratory, Porton Down, UK

## Abstract

The Ebola virus (EBOV) variant Makona (which emerged in 2013) was the causative agent of the largest outbreak of Ebola Virus Disease recorded. Differences in virus-host interactions between viral variants have potential consequences for transmission, disease severity and mortality. A detailed profile of the cellular changes induced by the Makona variant compared with other Ebola virus variants was lacking. In this study, A549 cells, a human cell line with a robust innate response, were infected with the Makona variant or with the Ecran variant originating from the 1976 outbreak in Central Africa. The abundance of viral and cellular mRNA transcripts was profiled using RNASeq and differential gene expression analysis performed. Differences in effects of each virus on the expression of interferon-stimulated genes were also investigated in A549 NPro cells where the type 1 interferon response had been attenuated. Cellular transcriptomic changes were compared with those induced by human respiratory syncytial virus (HRSV), a virus with a similar genome organisation and replication strategy to EBOV. Pathway and gene ontology analysis revealed differential expression of functionally important genes; including genes involved in the inflammatory response, cell proliferation, leukocyte extravasation and cholesterol biosynthesis. Whilst there was overlap with HRSV, there was unique commonality to the EBOV variants.

The evolution of Ebolaviruses of varying pathogenicity complicates assessment of the public health significance of novel filoviruses. Ebolaviruses are a diverse genus within the Filoviridae family. Five species have been characterised; from those non-pathogenic in humans to those with high case-fatality rates (CFR) ranging from 25–90%^1^,^2^. The recently isolated Ebola Virus (EBOV) Makona variant, from a clinical case of Ebola Virus Disease (EVD) in Macenta, Guinea, has been observed to have a varying CFR of 70% to 50% at later stages of the outbreak^1^. Detailed analysis revealed non-synonymous changes in the genome of the Makona variant compared with other previously identified variants of EBOV^3^,^4^, though the significance of many of these phenotypic changes is poorly understood.

Work *in vivo* using *Macaca fascicularis* as an animal model identified a potential shift in the pathogenesis of infection with EBOV Makona compared to other variants. Results showed a reduced pace of disease progression correlating with viral titre^5^. Differences exist in clinical data concerning disease course and mortality rates between the Makona variant and progenitors such as the Ecran variant of EBOV^1^,^3^. Analysis has also revealed differences in the ability of Makona and Mayinga to enter human-derived cell lines^6^. A more detailed assessment of mutations in the Makona glycoprotein demonstrated adaptation to entering human cells^7^. The possibility remains that infection with the Makona variant may activate or suppress different gene expression pathways when compared with variants of EBOV. Previous work to elucidate host cell responses induced by different filoviruses and EBOV *in vitro* and *in vivo* has revealed changes in the strength of activation observed in molecular pathways during infection^8^,^9^,^10^. Reston virus (RESTV) has never caused confirmed disease in humans and direct comparison of RESTV with EBOV identified molecular signatures associated with pathogenic filovirus infection^8^.

A full appreciation of global cell biology changes induced by the recent Makona variant has not been investigated. The profiling of cellular changes elicited by virus infection has been used previously to identify fundamental differences in cellular interactions, which occur between high and low pathogenicity variants. For example, with pandemic influenza H1N1 and a lower pathology variant^11^. A possible reason for differing pathogenicity observed in RESTV compared with EBOV infection may be reduced capacity to terminate virus replication through destruction of host cells. EBOV in contrast, is able to avoid initiation of apoptosis in cells to prolong infection and enhance production of viral progeny^12^,^13^. The implication being that significant differences in quantities of several host factors correspond to varying levels of pathogenicity observed in clinical infection^11^. Finding discernible differences between related viruses has the potential to be a powerful method to understand pathogenesis.

A direct comparison of cellular effects induced by different variants of EBOV and comparison to Makona would provide useful indicators to the biological properties of this virus. Therefore, the aim of this study was to define gene expression signatures associated with the Makona variant of EBOV compared to the 1976 Ecran variant by identifying significantly changed patterns of gene expression using RNAseq.

## Methods

### Mammalian cell culture and viruses

A549 cells were obtained from the European Collection of Cell Cultures (ECACC) maintained by Public Health England. A549 NPro cells are a stably transfected cell line expressing the Bovine Viral Diarrhoea virus (BVDV) N protein and was kindly provided by Prof. Steve Goodbourn (St George’s, University of London). The EBOV Makona variant was obtained from the European Mobile Laboratory as part of the response to the 2013–2016 West African outbreak, and is designated Ebola virus/H. sapiens-wt/GIN/2014/Makona-Gueckedou-C05. EBOV-Ecran was isolated during an outbreak in October 1976 (WHO International Commission, 1978) designated Ebola virus/H.sapiens-tc/COD/1976/Yambuku-Ecran. Both viruses used in this study were quantified by TCID_50_ by the Reed & Muench method^14^. Human respiratory syncytial virus (HRSV), strain A2, was used to infect A549 cells at an MOI of 0.5 TCID_50_ units/cell and cultured for 24 h, this was used as a comparator for pathway analysis. The HRSV strain used in this study is tissue culture adapted and was chosen as we and others have previously characterised this strain in A549 cells^15^. The Ecran variant of EBOV is an older isolate than the Makona variant with a lengthy passage history in Vero and Vero E6 cells. The Makona variant was recently isolated and used in this study after 2–3 passages and amplification in cell culture. A549 cells or A549 NPro were infected with EBOV-Makona or EBOV-Ecran at an MOI of 0.5 TCID_50_/cell at Containment Level 4 and total cellular RNA purified at 24, 48 and 72 hours post-infection using the Qiagen RNeasy Mini Kit. Total RNA was purified from cells infected with HRSV at 24 h post-infection. After this time point, for this virus, cells undergo syncytia formation. Replicates of A549 and A549 NPro mock infected controls were generated (n = 3) and all 72 h infections for Makona and Ecran were performed in duplicate.

### qRT-PCR analysis

RNA yields were measured with a Nanodrop 1000 (Thermo Scientific) and were normalised to 60ng/μl before addition to qRT-PCR. Primers and minor-groove binder probes were using previously described sequences^16^ to quantify the relative abundance of the EBOV genome. The reaction was set up with 900 nM of forward and reverse primers, 250 nM probe, 1x TaqMan Fast Virus Mastermix (Life Technologies) to a total volume of 20 μl and tested using the ABi 7500 (Life Technologies) at a thermal sequence of 50 °C for 5 minutes, 95 °C for 20 seconds followed by 40 cycles of 95 °C for 3 seconds and 60 °C for 30 seconds Samples were tested in triplicate and results analysed at a threshold value of 0.2. For gene-specific RT-PCR profiling SYBR Green gene specific PCR assays were obtained as validated commercially available assays (Qiagen). RNA was treated with RNase free DNase (Promega) and cleaned up using the RNEasy MinElute Kit (Qiagen). RNA was equalised between all samples tested using nanodrop measurement (Thermo Scientific). Assays were performed in two stages, with the reverse transcription (RT) step performed with the RT2 First Strand kit (Qiagen) and the gene specific assay performed with gene specific primers (Qiagen) and the RT2 SYBR Green qPCR Mastermix (Qiagen), melt curves were performed to verify specificity of primer sets.

### Western Blot Assays

Western blots were performed on protein extracts collected in Laemlli Buffer (Sigma) supplemented with an additional 10% SDS to comply with local controls for the removal of samples from containment level 4. Samples were heated at 95 °C for 10–15 minutes prior to removal to containment level 2. Protein lysates were loaded on to Novex 4–12% gradient acrylamide gels (Life Technologies). The gels were run in a Novex X-2 vertical gel tank for 50 minutes at 200 V in MOPS buffer (Life Technologies). PVDF blots were performed using the IBlot semi-dry blotting system (Life Technologies) and blots blocked for minimum 2 h at room temperature with non-fat dried milk (NFDM; Sigma) in Tris-Buffered Saline–Tween20 (TBST; Sigma). Blots were rinsed in TBST and primary antibody staining performed overnight with the IRF-3 and GAPDH specific rabbit polyclonal antibodies (Millipore). Secondary antibody staining was performed as per antibody instructions using goat anti-rabbit antibody conjugated to HRP. Luminescence assay was performed with ECL Prime reagent (GE Healthcare) after a 5 minute incubation and imaged in a GeneSys Gel Imaging System (GeneSys).

### RNASeq

The RNA samples were DNase treated and prepared for Illumina sequencing on a HiSeq 2500as described in previous work^17^. Final libraries were pooled in equimolar amounts using the Qubit and Fragment Analyser data. The quantity and quality of each pool was assessed by the Fragment Analyser and subsequently by qPCR using the Illumina Library Quantification Kit from Kapa on a Roche Light Cycler LC480II according to manufacturer’s instructions. The template DNA was denatured according to the Illumina cBot protocol and loaded at 12 pM concentration. To improve sequencing quality control 1% PhiX was spiked-in. The sequencing was performed on three lanes of an Illumina HiSeq 2500 with version 4 chemistry generating 2 × 125 bp paired end reads.

### Bioinformatics Analysis

Briefly, base calling and de-multiplexing of indexed reads was performed by CASAVA version 1.8.2 (Illumina) to produce 30 samples from the 5 lanes of sequence data, in fastq format. The raw fastq files were trimmed to remove Illumina adapter sequences using Cutadapt version 1.2.1^18^. The option “-O 3” was set, so the 3′ end of any reads which matched the adapter sequence over at least 3 bp was trimmed off. The reads were further trimmed to remove low quality bases, using Sickle version 1.200 with a minimum window quality score of 20. After trimming, reads shorter than 50 bp were removed. If both reads from a pair passed this filter, each was included in the R1 or R2 file. If only one of a read pair passed this filter, it was included in the R0 (unpaired reads) file. The reference genome used for alignment was the human reference genome assembly GRCh38. The reference annotation used was GRCh38.77. The annotated file contained 63,152 genes. R1/R2 read pairs were mapped to the reference sequence using TopHat2 version 2.1.0^19^, with the mapper Bowtie2 version 2.0.10^20^.

### Differential Gene Expression Analysis

Viral mapped reads were further analysed using Cufflinks with the CuffDiff algorithm to calculate fragments per kilobase per million (FPKM) and viral mRNA levels determined for each virus at each time point. Mapped reads to the human genome were also analysed using EdgeR v. 3.3^21^ and DESeq to calculate normalised counts per million (CPM) differential gene expression comparing infected conditions with mock infected data sets. Ingenuity Pathway Analysis (IPA) was used to perform gene ontology and pathway analysis.

## Results

Two different EBOV variants were investigated in this study; the EBOV Makona isolated from the 2013–2016 West Africa outbreak and the progenitor virus, EBOV Ecran, isolated in 1976. The effect of the two EBOV variants on the host transcriptome of A549 cells was investigated. A549 cells were chosen for this study because they produce a robust innate response to virus infection and have been used to study the innate cell response to a number of different viruses^11^,^22^,^23^,^24^,^25^, including EBOV^26^. A549 cells are also permissive to HRSV infection. HRSV was used as a comparator in downstream experiments as this virus has a similar genome organisation and replication strategy to the EBOV variants.

### EBOV variants displayed similar replication rates in A549 cells

**Figure 1:**
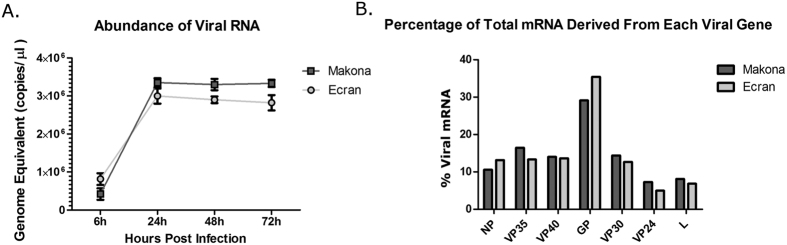
Viral replication rates and mRNA abundance. (**A**) Graph showing the genome abundance of either Makona or Ecran at 24 h, 48 h or 72 h post-infection in A549 cells. Replicate infections were tested by qRT-PCR to estimate viral genome abundance. The data is shown as genome equivalent copies per μl of RNA extract. Bars represent standard deviation. The y-axis is shown in the log10 scale to aid interpretation. (**B**) Histogram showing the relative abundance of viral mRNAs at 24 h post-infection of either Makona or Ecran in A549 cells. Sequence reads were assembled into transcripts and mapped to viral mRNA sequences using Cufflinks 2.0 in the Galaxy analysis environment. The genes were arranged by order along the genome and normalised to both gene length and total mapped reads. The data shows the percentage of total assembled and mapped reads which aligned to each gene for Makona and Ecran.

Both the Makona and Ecran variants were titred by TCID_50_ and cells infected with an equal MOI (0.5 TCID_50_/cell). The abundance of genome was compared by qRT-PCR analysis (Fig. 1A) and analysed using a repeated measure General Linear Model with Greenhouse-Geisser correction. Duplicate infections were performed in parallel for each virus and qRT-PCR assays performed in triplicate for statistical analysis. This analysis showed no significant difference in the abundance of the genomic RNA level between Makona and Ecran at 24 h, 48 h and 72 h post infection, suggesting that replication was equivalent in this cell line, and any observed differences in host cell gene expression would not be dependent on differences in the growth kinetics of the two EBOV variants. Therefore, total cellular RNA was prepared at 24, 48 and 72 h post-infection from the Makona, Ecran and mock-infected cells. Transcriptomic analysis of infection was performed at these time points post-infection, representing when progeny virus was first released at 24 h until 72 h, where cytopathic effects become apparent.

The abundance of viral mRNA was compared between the variants to confirm the qRT-PCR analysis of progeny virus production and examine whether the synthesis of EBOV mRNAs corresponded to a transcription gradient or differed in pattern or abundance between variants. For members of the *Mononegavirales*, to which EBOV belongs, synthesis of mRNAs has been postulated to reflect a transcription gradient^27^, where genes transcribed from the genomic 3′ end are more abundant than genes located at the 5′ end. However, recent work has suggested the transcription pattern for viral mRNA transcribed by Hendra virus and HRSV^17^,^28^, both members of the *Mononegavirales*, is not necessarily related to the position of a gene along the viral genome.

Mapping of EBOV genes was conducted as previously described for our analysis of HRSV in cell culture^17^, EBOV in a guinea pig model of infection^29^ and analysis of EBOV Makona genome evolution in outbreak samples from Guinea^4^. The abundance of the different viral mRNAs was calculated by mapping RNASeq data to the EBOV genome, separating mRNAs by open-reading frame and normalising gene reads to viral gene length (Fig. 1B). The data indicated that there were no significant differences in gene expression patterns between the Makona and Ecran variant. For both variants, the abundance of the NP mRNA was greater than that of L mRNA, however the abundance of the viral mRNAs transcribed between these genes was not linear. This suggested that factors other than the polarity of transcription may have affected viral mRNA abundance with EBOV. Note that the abundance of GP mRNA shown did not distinguish between reads mapping to sGP or full length GP or any other variants of this transcript.

### Differential gene expression analysis of infected A549 cells revealed differences between Makona and Ecran in the host response

**Figure 2:**
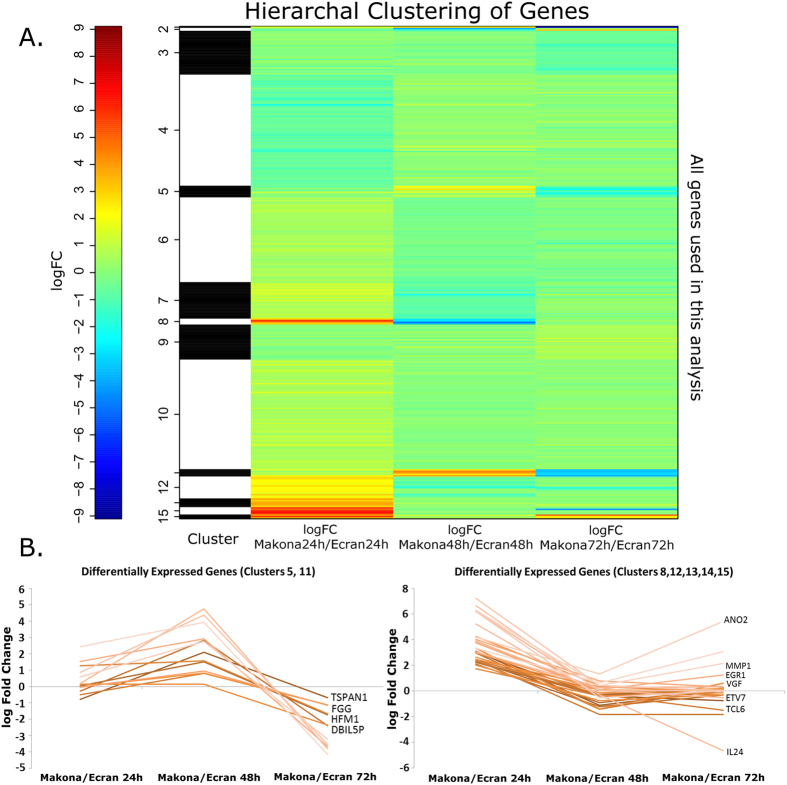
Hierarchal clustering. (**A**) Hierarchal clustering of differentially expressed genes in A549 cells infected with either Makona or Ecran (white/black shading) at 24 h, 48 h and 72 h post-infection. Red indicates a large fold increase and blue indicates a large fold decrease in mRNA abundance for that gene. (**B**) Genes displaying a significant difference in abundance by comparing 24 h/48 h and 48 h/72 h are shown in graphs as a parabolic curve. The graph on the left hand side shows genes with a similar pattern of abundance, which aligned to clusters 5 and 11. The graph on the right shows clusters 8, 12, 13, 14 and 15, which again had a similar pattern of abundance over the time points analysed. The selected transcripts are from genes whose mRNAs encode inflammatory modulators and produce non-coding RNA.

**Figure 3:**
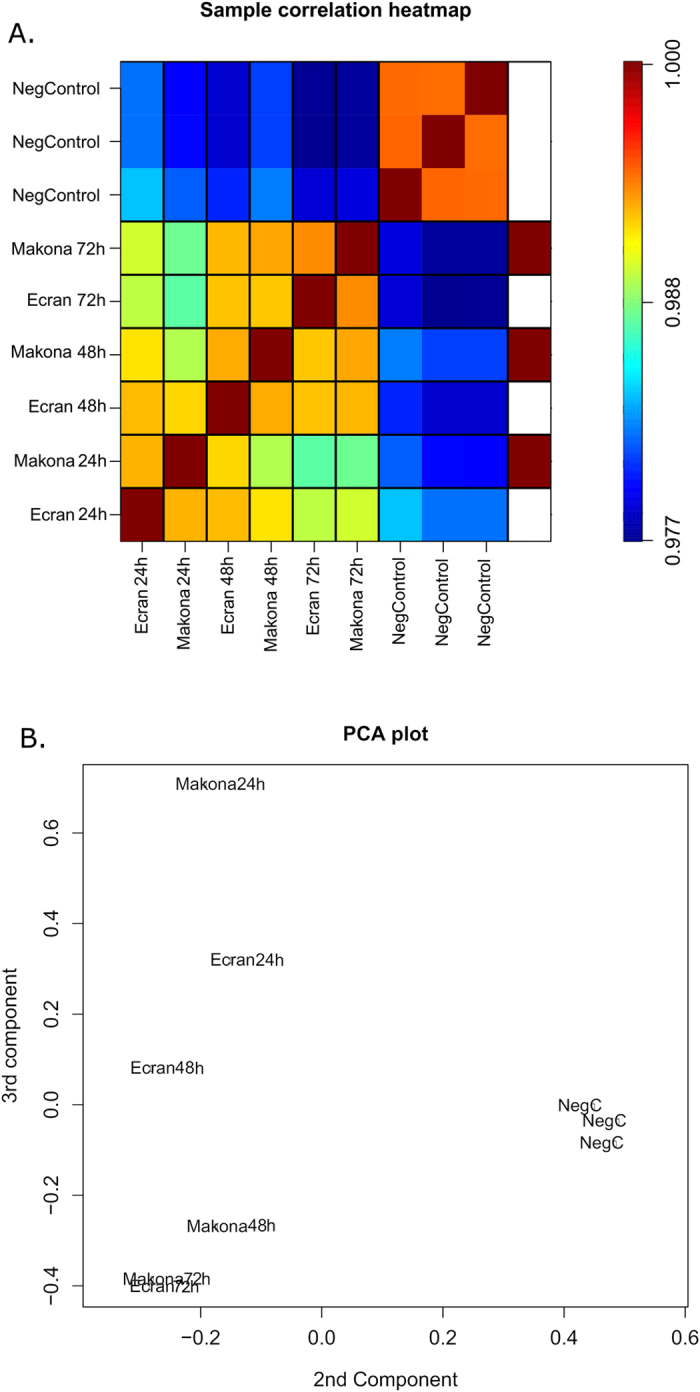
Sample Correlation. (**A**) The correlation heatmap illustrates the relationship between each dataset compared to every other dataset. Red indicates a close correlation while blue indicates a more distant correlation in the data. This displays the correlation of samples increasing over time, but becoming more distant from mock infected. (**B**) Additionally a Principle Component Plot was generated to model the relationship between each dataset. This shows that data collected from mock infected (n = 3) and infected cells can be clearly separated by the 2^nd^ and 3^rd^ component. This also shows that 24 h is the most divergent time point. Thus 24 h post-infection became the focus for further analysis.

To investigate host gene expression in EBOV infected cells, non-viral sequence reads were mapped to the human transcriptome. Data was then modelled following a negative binomial (NB) distribution. Levels of mRNA were normalised to account for size factors and general linear models (GLM) were employed^30^. The TMM (trimmed mean M-values) method was used. Mock infected replicates of either A549 or A549 NPro (n = 3) were grouped and analysed in EdgeR to estimate common, trended and tag-wise dispersion in a control group. The control group dispersion was used to model and calculate false-discovery rates (FDR) for infected samples, which were analysed as single samples of each condition. A dispersion plot was generated to show dispersion against log2CPM (Supplementary Data 1A) and FDR corrected p-values were calculated. Data was then selected for significant fold change and counts per million (CPM) (Supplementary Data 1B). Genes were separated into those showing increased or decreased abundance compared to their expression in mock infected cells (Table 1). The fold changes in cellular mRNA abundance were calculated for cells infected with Makona and directly compared to those infected with Ecran; these were visualised by Volcano Plot (Supplementary Data 1C) and Hierarchal Clustering (Fig. 2A). This analysis indicated most gene expression differences were apparent at 24 h post-infection. Several clusters displayed significant changes between time points and were grouped by positive or negative change from 24 h to 48 h (Fig. 2B). These charts display patterns of gene expression which changed throughout the time course experiment; governing multiple genes including *IL24, TSPAN1, FGG* and *MMP1*. The data was assessed to evaluate the similarities between each condition; a correlation plot was generated (Fig. 3A) and a principal component analysis (PCA) was performed (Fig. 3B). This analysis confirmed that clear differences in the abundance of mRNA in virus infected cells occurred by 24 h. Therefore, the 24 h data was further analysed by grouping changes in transcript abundance for pathway analysis.

### Makona and Ecran displayed patterns of pathway induction which were distinct from HRSV

**Figure 4:**
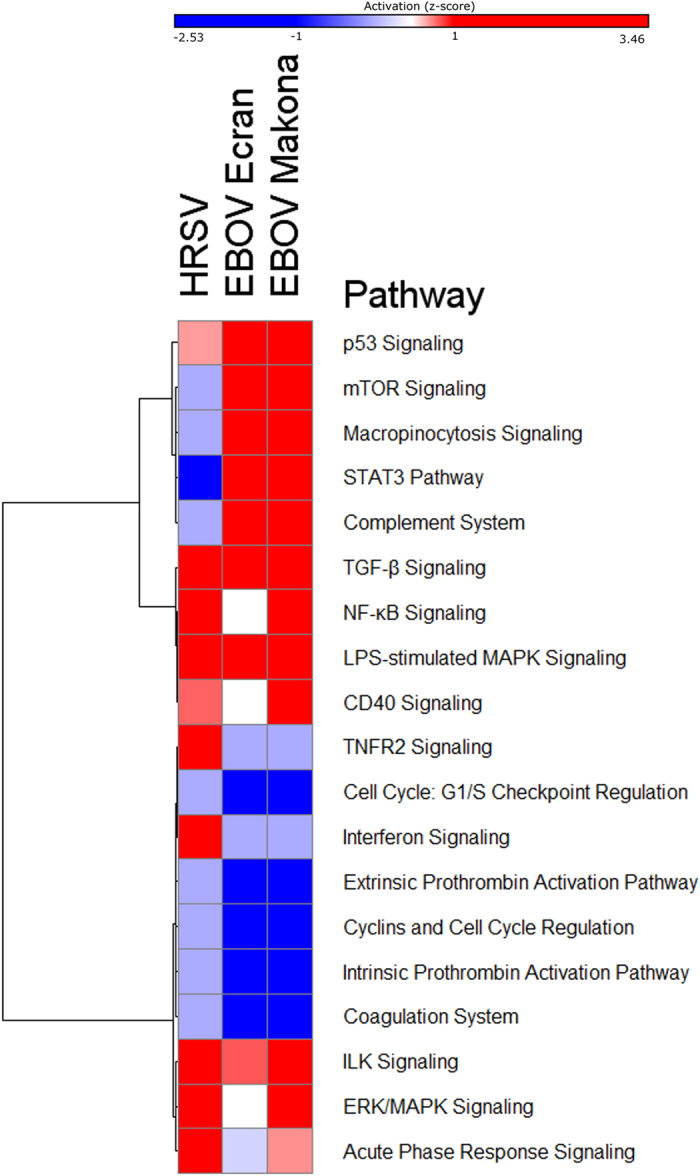
Comparison of pathways induced by HRSV and EBOV infection. Pathway activation scores for HRSV and Makona and Ecran were compared to identify patterns of pathway activation specific to EBOV infection. Pathway activation score (z-score) was calculated using the cumulative increase or decrease in abundance of transcripts from all genes which mapped to that functional pathway. A scale bar is provided with colour partitions at z-score of -1 or below indicated by an intense blue colour or 1 or above indicated by red. The results are displayed as a heat map indicating activation of each pathway for the three viruses analysed; HRSV, Ecran and Makona.

Transcriptome changes in cells infected with the variants of EBOV may have reflected generic responses to viral infection, rather than being specific for EBOV. To investigate and control for this, A549 cells were infected with HRSV at an MOI of 0.5 PFU/cell and the total RNA purified at 24 h post-infection and analysed by RNASeq. The EBOV and HRSV data were then used to calculate pathway activation scores in IPA (Z-scores); reflecting the cumulative patterns of increased/decreased abundance of gene transcripts aligned with a pathway and thus the pathway activation state (Fig. 4). This analysis revealed pathways with both similar and dissimilar patterns of induction in the EBOV variants. Similar pathways included those governing exocytosis, inhibition of metalloproteases, and the LPS-stimulated MAPK pathway. Differences were observed in pathways governing cell cycle regulation and coagulation, which were decreased more in EBOV infection compared to HRSV making this a defined characteristic of EBOV infection compared to HRSV in this cell culture system. Conversely the TNFR2 and interferon signalling pathways showed higher activation in HRSV infection but not in EBOV infection; the latter is consistent with previous observations that EBOV proteins are able to disrupt interferon signalling in cell culture^31^,^32^. The p53 signalling pathway, STAT3, mTOR and complement signalling had a higher activation score in EBOV infection than in HRSV. Induction of all canonical pathways in Makona and Ecran infected cells was assessed and visualised by hierarchal clustering (Supplementary Data 2); showing that most pathway induction was similar for both Makona and Ecran. This analysis also indicated that ~85% of pathways with more than a single aligned gene showed activation (high activation score) while only ~15% showed deactivation (low activation score). All genes with a p-value less than 0.01 were aligned to canonical pathways, the top 10 pathways to which these genes aligned is shown in Table 2, indicating that the most significant gene pathways included EIF2 signalling, mitochondrial dysfunction pathways and cholesterol biosynthesis pathways.

### Analysis revealed stimulated Interferon Stimulated Genes (ISGs) common to all tested viruses and those unique to EBOV

**Figure 5:**
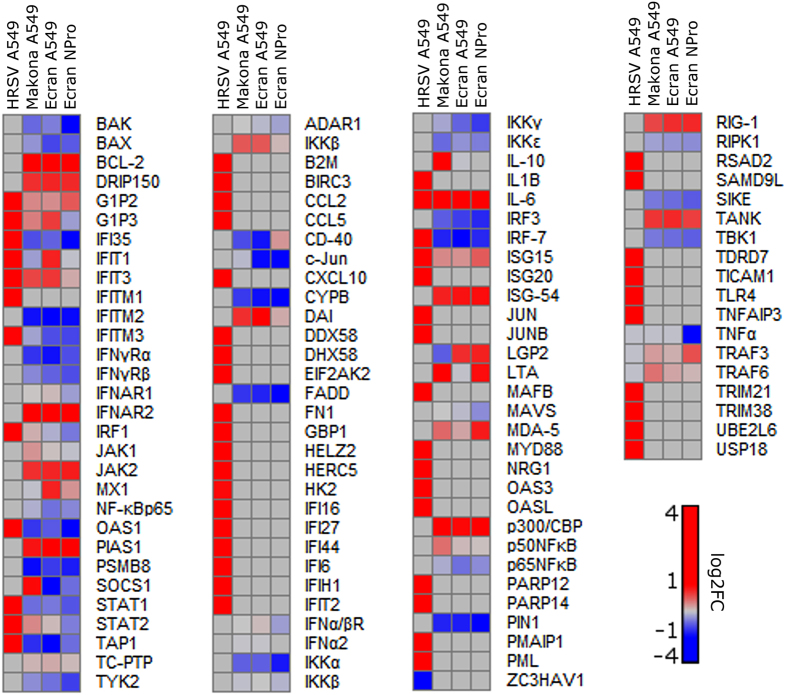
Comparative analysis of transcript abundance in A549 and A549 NPro cells infected with either Makona or Ecran. Heat maps displaying the log2 fold change in abundance of gene transcripts regulated by IRF-3. Gene expression changes are shown for Makona infected A549, Ecran infected A549, HRSV infected A549 or Ecran infected NPro cells. A scale bar is provided with partitions at log2 fold changes of 1 and −1 to aid interpretation. An intense red colour indicates an increase in mRNA abundance of 1 log2 fold change or above, and blue indicates a decrease of −1 log2 fold change or below. Grey indicates no significant change.

**Figure 6:**
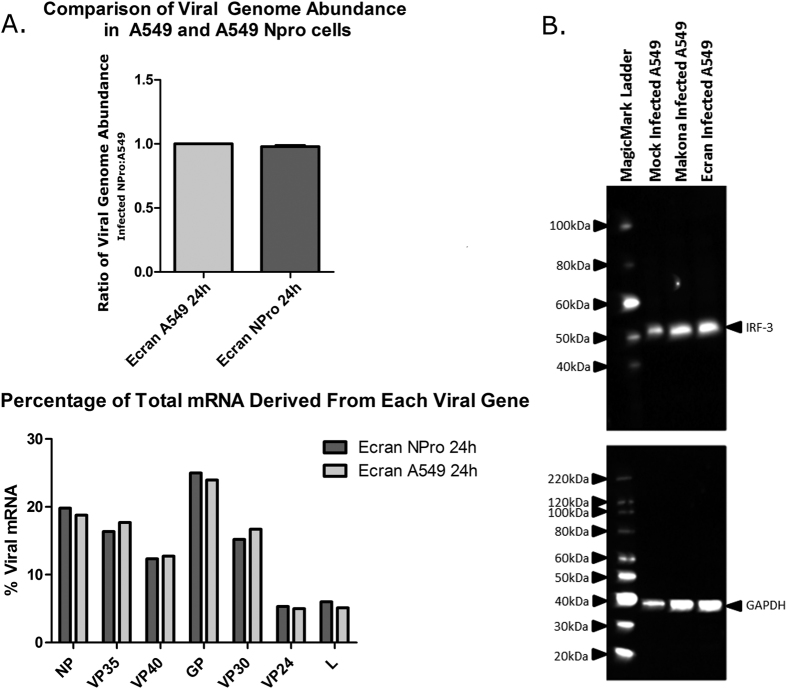
Analysis of viral genome and mRNA abundance and validation of IRF-3. (**A**) qRT-PCR was used to measure the abundance of EBOV GP in either A549 cells or the A549 NPro cell line and (**B**) RNAseq data of viral transcript abundance was used to compare the replication of the Ecran variant in both A549 and A549 NPro cells. Abundance of viral genome copies intracellularly were measured for the 24 h time point in both cell lines. Fold change in genome abundance was calculated. For RNAseq, reads corresponding to viral genes were mapped to the viral genome and percentage of reads per gene calculated. (**B**) A western blot was performed to visualise IRF-3 in A549 cells infected with either Makona or Ecran to identify any difference in levels of available IRF-3, which may explain downstream regulatory activity changes. IRF-3 was probed using commercially available rabbit anti-IRF-3 and anti-rabbit horseradish peroxidase conjugated secondary antibody. GAPDH was used as a loading control to aid interpretation of the band intensity for IRF-3. Note additional SDS was added to cell lysate to render EBOV-infected material safe to transfer from higher to lower containment.

The difference in activation of interferon signalling induced by HRSV compared to the EBOV variants was further investigated to assess whether Makona and Ecran infection resulted in identical levels of interferon activation. Previous work showed inhibition of IRF-3 and STAT1 by two EBOV proteins; VP35 and VP24 were important contributors to viral virulence and rapid viral growth in cell culture^33^,^34^,^35^. To investigate possible differences in the abundance of ISGs between Makona and Ecran infection A549 cells were used in which interferon signalling had been blocked (A549 NPro cells)^36^. A549 NPro cells constitutively express the N protein of bovine viral diarrhoea virus (BVDV), which binds to IRF-3 and signals for degradation via the proteasome^36^. This results in failure to activate downstream gene expression in response to type 1 interferon. This recombinant cell line was infected with the Ecran variant and gene expression changes compared with those of interferon competent A549 cells. Expression patterns of ISGs induced by Makona, Ecran or HRSV were compared with EBOV infected A549 NPro cells (Fig. 5). This comparison revealed most ISG transcripts did not significantly change in A549 cells infected with either Makona or Ecran infection or in A549 NPro infected with Ecran. The change in abundance of many ISG transcripts appeared unique to HRSV. *IL-10, TRAF6, STAT2, c-Jun* and *LTA* all showed expression levels in Makona infected cells different from those of Ecran but similar to that of Ecran infected A549 NPro cells. LTA is a TNF-α family cytokine with potent immunomodulatory activity^37^. The abundance of IRF-3 transcript was not significantly different between Makona and Ecran infected A549 or A549 NPro cells. Transcripts encoding the apoptotic regulators *BAK* and *BCL-2* were increased in abundance in infected NPro cells compared to infected A549 cells, whilst transcripts encoding *IFI35, IFIT3* and *IFNAR* were all decreased in abundance. Perturbation of interferon signalling should make cells more permissive to viral infection. To assess whether EBOV Ecran displayed accelerated replication in A549 NPro cells, qRT-PCR was performed to assess intracellular viral genome abundance (Fig. 6A) and to assess differences in the relative abundance of viral mRNA corresponding to each viral gene. This analysis revealed that there was no difference in viral replication rates between A549 and A549 NPro and that patterns of viral gene transcription were similar. IRF-3 protein abundance was investigated by western blot shown in Fig. 6B. GAPDH was probed as housekeeping protein, and allowed comparison of varying protein concentration between mock and infected samples, and interpretation of results was adjusted appropriately on this basis. This showed that the abundance of IRF-3 was similar in Ecran and Makona infected A549 cells.

### Cholesterol biosynthesis is activated in EBOV infected cells

For both Ecran and Makona, pathway analysis of the transcriptome changes in A549 cells indicated activation of the cholesterol biosynthesis pathways (i.e. p-value of 1.14 × 10^−8^ for Makona and 5.26 × 10^−9^ for Ecran). Related to this was the increased abundance of the Niemann-Pick disease, type C1 (NPC1) transcript in virus infected cells (log2 2.265 and 1.847 increase for Makona and Ecran, respectively). NPC1 has been identified as a receptor for EBOV entry^38^ and its normal function in cells is in the intracellular transport of cholesterol to post-lysosomal destinations^39^. In contrast NPC1 transcript abundance was unchanged in HRSV infected A549 cells compared with Mock, indicating that increased NPC1 gene expression is not a generic response to viral infection. Upstream activators of NPC1 identified in our analysis included transcription factor 7 like 2 (TCF7L2), which increased in log2 fold abundance in Makona and Ecran infected cells by 2.789 and 2.819, respectively.

### Makona displayed significant differential expression of several interconnecting genes, compared to Ecran

**Figure 7:**
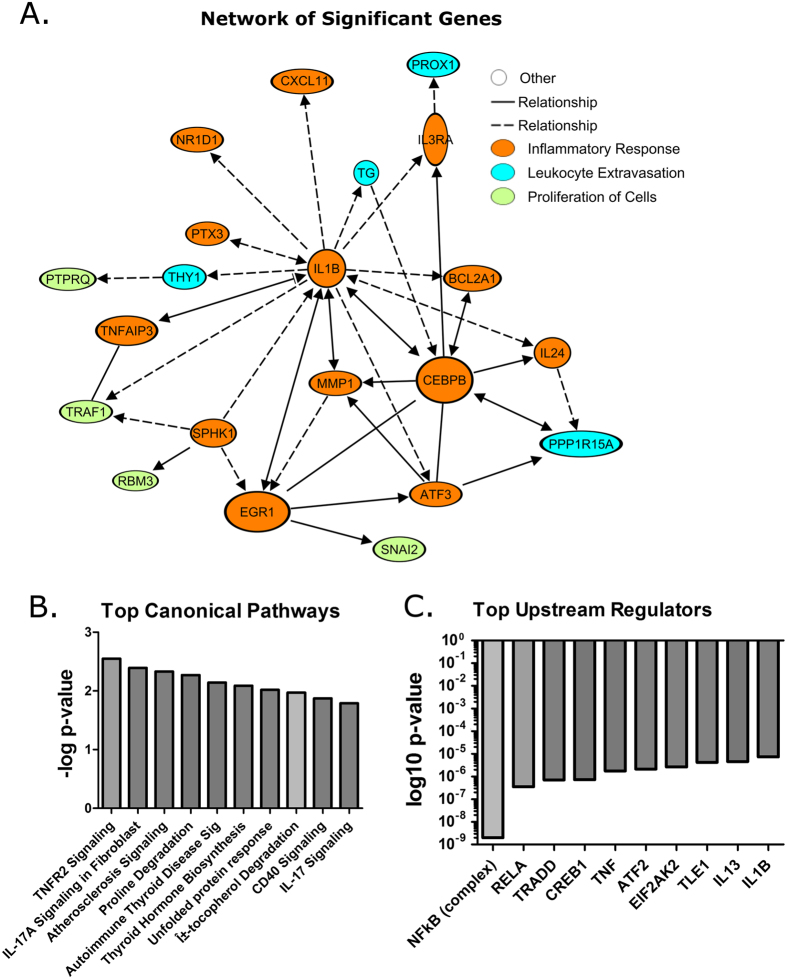
Significant differentially expressed genes. (**A**) Functionally annotated network of connected and functionally relevant genes, only those with direct or indirect connections are shown which were both significantly different in abundance in cells infected with Makona compared to Ecran and significantly changed compared to mock infected cells. Five functional networks are displayed and genes coloured to reflect the network in which they belong. The network with the greatest gene numbers is the inflammatory response, cell death and survival network. (**B**) Top canonical pathways to which the genes shown align is displayed in the histogram ranked according to p-value. (**C**) The predicted upstream regulators arranged in order of p-value, which regulate expression of the genes is shown.

**Figure 8:**
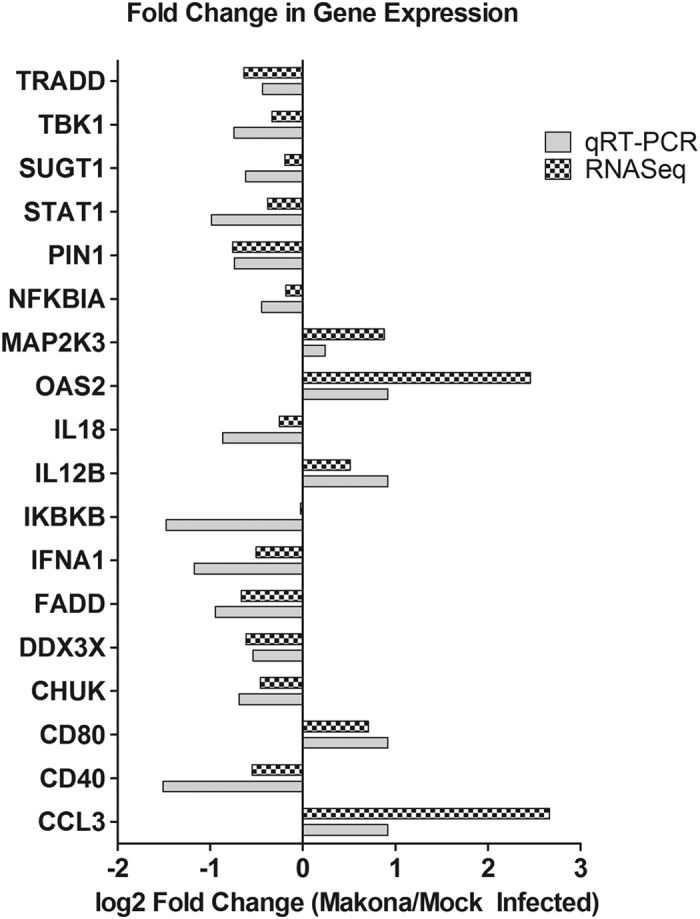
Confirmation of selected cellular gene expression in Makona infected cells by qRT-PCR. A549 cells were infected with the Makona variant of EBOV in duplicate and RNA isolated at 24 h post infection. Mock infected A549 RNA and Makona infected A549 RNA were subjected to 2-step gene specific RT-PCR analysis, with the RT step performed and then real-time PCR performed with SYBR green. Melt curve analysis was used to confirm specificity of each gene specific assay. Log2 Fold change was calculated using mean CT values of each gene and the the deltadeltaCT method was employed, calculated gene expressions werenormalised to a panel of housekeeping genes including HPRT, GAPDH, B2M and B-actin. Log2 fold changes are displayed against each gene tested as either below -1 or above 1 fold change compared with mock infected A549 cells. Paired with each qRT-PCR gene expression result, is the result of RNASeq analysis to allow easy comparison and data validation.

The abundance of transcripts corresponding to ISGs and IRF-3 regulated genes appeared similar for both variants of EBOV, and therefore broader analyses of significantly differentially expressed genes were performed. Figure 7A shows those genes that were most significantly changed in abundance in Ecran infected cells compared to Makona infected cells. Only those genes with direct interactions are displayed in Fig. 7A. These genes have been coloured to reflect alignment to functional networks. The largest numbers of genes are grouped under inflammatory response and proliferation of cells, with the rest grouped under leukocyte migration. An expanded view of all significantly changed genes irrespective of network interactions is shown in Supplementary Data 3. The largest numbers of genes are grouped under inflammatory response, cell death and survival, with the rest grouped under a further 5 functional networks broadly categorised under Organismal Injury. The abundance of several transcripts were previously found to be significantly changed in a previous analysis of EBOV and Marburg virus (MARV) *in vitro*^8^. In our analysis, 21 transcripts encoded proteins which possessed nuclear activity, mostly as transcriptional activators or co-factors including *EGR1* and *ATF3. IL-24* was also increased in abundance, and this is a potent immuno-modulator. Figure 7B displays the canonical pathways to which these genes best align. This analysis revealed common predicted upstream regulators of the genes shown (Fig. 7C), the top predicted regulators being TNF, EIF2 regulators and IL1B. To confirm the change in abundance of transcripts encoding products involved in antiviral activity, the infection of A549 cells with Makona was repeated in duplicate, and the abundance of these transcripts compared using gene specific RT-PCR arrays (Fig. 8). The assays revealed similar patterns of changes in transcripts corresponding to antiviral genes, including *CXCL8, IRAK1* and *IRF5*.

## Discussion

This study compared viral gene expression and the host response at a cellular level between the Makona isolate of EBOV and the potential progenitor Ecran variant. Other studies have used genomic approaches to identify markers associated with severe disease presentation and lethality with EBOV^9^. To characterise the potential cellular changes to EBOV infection and to determine whether these were different between two EBOV variants, the growth of the one modern and the progenitor virus was compared in a cell culture system. This was coupled to RNAseq to examine the overall levels of mRNA abundance at different time points. The cell culture system used was the A549 cell line and these are human epithelial in origin. One advantage of using such a cell line is the uniformity of the experimental system for transcriptomic analysis. However, caution should be used when interpreting results from such systems as these cells have a modal chromosome number of 66, and therefore do not resemble *ex vivo* or *in vivo* cells from humans. However, the A549 cell line has a robust immune response and has been used extensively for researching virus/host interactions, and allowed a direct comparison between EBOV and HRSV. Thus discriminating between potential generic anti-viral effects and ones intrinsic to a specific virus.

Differences in the host response to infection with EBOV Makona or Ecran may have been due to different replication kinetics. Therefore, the replication of Makona and Ecran was compared in A549 cells by measuring genome copies present in the cell at 24, 48 and 72 h post-infection by qRT-PCR. The data indicated that both variants produced progeny virus at equivalent rates at the time points assessed. This was also reflected in the number of reads mapping to each viral mRNA, providing a comparison of the amount of viral RNA present in the cells at the time point assayed. This analysis confirmed that any changes in the abundance of host transcripts between the two variants were not due to differential kinetics of viral replication.

Interestingly, the abundance of viral mRNA did not conform to a precise linear transcription gradient, as is generally assumed in the *Mononegavirales*^40^. For EBOV in cell culture, there appeared to be a clear delineation in viral mRNA abundance at the VP30/VP24 gene junction. This may be due to the VP30-VP24 intergenic region being longer than in similar viruses. Reporter gene studies suggested that the length of this region can affect the frequency of transcriptional re-initiation^41^. There was greater abundance of viral mRNA attributable to GP expression compared to other viral mRNAs, although it was not possible to assign reads mapping to the different isoforms of GP. Recently, RNAseq analysis of the abundance of viral RNA in cells infected with Hendra virus or HRSV indicated that transcriptional profiles do not fit a precise model of a linear gradient and other factors such as RNA stability maybe important^17^,^28^. Alternatively, the abundance of viral RNAs as indicated by RNAseq may be due to properties of the algorithms used to assign mapped reads, although manual assignment suggested this was not the case^17^.

The analysis of global host gene expression changes revealed that EBOV Makona and Ecran variants elicited more diversified gene expression changes at the 24 h time point in cell culture infection compared to later time points. Infection with EBOV triggered a cascade of phenotypic changes in the cell designed to inhibit infection and preserve cell integrity^42^. The observation that differences between the tested variants was clearest at 24 h implies that expression changes at this time point were determined by viral phenotype rather than a general cellular response. Patterns of expression converge at 48 h and were almost identical at 72 h post infection. At 72 h, cytopathic changes were more apparent and characterised by a worsening of cellular condition and adverse cellular phenotypic changes^43^,^44^. To better deduce the effects attributable to differences in viral phenotypic differences, the 24 h samples were selected for further analysis. Canonical pathway analysis indicated that infection with the Makona variant appeared to result in suppression of the EIF2 signalling pathway, a pathway influencing cell survival^45^, and mitochondrial dysfunction, a pathway connected to apoptotic processes.

The changes in transcript abundance in A549 cells infected with EBOV Makona correlated with the changes observed in a mouse model of EBOV Mayinga infection. In this model, several gene signatures associated with lethality were identified^9^. Indeed, patterns of gene expression induced by Makona infection of A549 cells correlated better with this signature of lethal infection in mice than the pattern induced by Ecran infection of A549 cells. Examples of abundance changes of transcripts in Makona infected A549 cells included *SOCS2, IL-6, PLAU, MMP-3*, -*9* and -*13*, and *TIMP1*, which are involved in acute phase signalling, chemotaxis and leukocyte extravasation. The acute phase response is associated with liver damage, a characteristic observed in patients infected with the Makona isolate^46^ and whose gene expression signatures were identified in a differential gene expression analysis of blood from patients with acute EVD^47^.

Comparing patterns of pathway activation in EBOV and HRSV infection showed that several cellular pathways had dissimilar levels of activation. Amongst these was the cell cycle regulatory pathway, with implications for the lifecycle and replication of EBOV. Cell cycle deregulation and changes in the abundance of cell cycle regulatory gene transcripts (and proteins) have been observed in HRSV in cell culture^15^,^48^,^49^,^50^, and this was shown to enhance virus infection^25^,^50^. In comparison, cell cycle deregulation appeared even more pronounced in EBOV infected cells. There was differential activation of interferon and p53 signalling pathways compared with HRSV. The p53 signalling pathway is heavily involved in determination of the fate of a cell and has been implicated in mediating cell cycle arrest during viral infection^49^.

The interferon signalling pathways have been studied in EBOV infection in cell culture, leading to the discovery that the viral encoded proteins, VP35 and VP24 are able to disrupt interferon signalling^8^,^10^,^44^. However, *in vivo* analysis of blood taken from patients with acute EVD from Guinea suggested that there was a robust interferon response^47^. Disruption of these pathways would be predicted to result in reduced cellular resilience to viral infection. We investigated whether the activity of IRF-3 was differentially regulated by EBOV Makona, Ecran or HRSV. Additionally, we sought to determine whether other antiviral factors other than those regulated by interferon affected viral replication by infecting the interferon signalling deficient A549 NPro cell line with Ecran (Fig. 5). Our data suggested that genes under the regulatory power of IRF-3 were expressed higher in HRSV infection than EBOV Makona or Ecran infection of A549 cells. Amongst these, *IFI35, OAS1, STAT1, TAP1* and *IRF-7* gene transcripts were all increased in abundance in HRSV infected A549 cells compared with mock infected A549 cells, and decreased in abundance in EBOV Makona and Ecran infected A549 cells compared with mock infected A549 cells. *BAK, BAX, FADD, IKKα* and all showed decreases in abundance in Makona or Ecran infected A549 and NPro cells compared with mock infection. *IL-6* expression was also induced in HRSV, Makona and Ecran infection of A549 cells and in Ecran infection of A549 NPro cells. IL-6 was evaluated in the *Macaca fascicularis* model system comparing the pathogenesis of EBOV Mayinga variant with Makona. This experiment indicated that IL-6 concentration in serum reached similar levels for both variants at pre-defined end points but rose more quickly in Mayinga infection^5^. IL-6 gene transcripts were also shown to be activated in patients with acute EVD and infected with the Mayinga variant^47^,^51^. Although most quantified interferon stimulated gene expressions correlated well between Ecran and Makona in cell culture some differences were identified. For example, the increased abundance of the inflammatory mediators, *IL-10* and *SOCS1*, in Makona infected cells. More importantly, comparison of Ecran infected A549 cells with Ecran infected A549 NPro cells showed that disruption of interferon signalling in the A549 NPro cell line did not enhance the replication rates of Ecran. The implication is that interferon has little impact on viral growth due to the potent modulatory activity of EBOV virulence proteins.

Activation of cholesterol pathways in infected cells was shown to be common in both EBOV variants but not found in HRSV. Cholesterol has been shown to be important for detachment of infected cells and this was induced by membrane anchored EBOV GP^52^. Cell detachment during EBOV infection results in the loss of the endothelial barrier in blood vessels and leads to vascular leakage. As a result cholesterol-reducing treatments have been proposed as part of treatment regimes^52^.

Amongst the most significant findings of this study were the observed differences between Makona and Ecran induced expression of genes linked to the inflammatory response, proliferation of cells and leukocyte extravasation. Less than 1% of the entire mRNA profile identified in this study showed significant differences between Makona and Ecran, suggesting the phenotypic differences between these viral infections are relatively small. Several gene transcripts which were previously shown to change in abundance in cells infected with the Mayinga variant of EBOV were identified in this study, including *TRAF1, PLB1, CTU1, TNFAIP3, PTX3, ATF3* and *CEBPB*^9^,^53^. IL-24 has immune-modulatory effects and expression has been shown to negatively impact Influenza A infection^54^. *EGR1* is differentially expressed as well as *ATF3*, both are regulatory factors governing cell activation in response to stress. Aligning genes to canonical pathways (Fig. 7B) showed TNF and immune response associated pathways amongst the top 10 pathways, and prediction of upstream regulators based on downstream activation data shows that NFκB, TRADD, RELA, TNF, EIF2AK2 and IL1β may display differing levels of activity in Makona infection compared with Ecran (Fig. 7C). *TNFAIP3*, a negative regulator of TNF induced apoptosis and *TRAF1*, a regulator of NFκB, JNK and a negative regulator of TNF mediate apoptosis showing significant change in transcript abundance. Interferon induced genes were also differentially expressed. compared with Ecran infection. IL1β is a pro-inflammatory regulator with stimulates a range of cellular innate immune response events. The abundance of *IL1β* transcript is significantly different comparing Makona with Ecran but does not appear significantly changed compared to mock infected, and thus the biological implications of this differential expression are unclear. Taken together our data suggested subtle differences in the activity of pathways governing cell response to viral infection including TNF, EIF2, Type 1 interferon and the NFκB complex associated pathways comparing Makona with Ecran in A549 cells. Additionally, we show that the known capabilities of EBOV to dysregulate interferon stimulated genes were not different between Makona and Ecran, and that only a small fraction of genes appear differentially expressed comparing these two variants.

## Conclusion

Small changes in pathway activity could result in profound implications for the cell. Overall, there was reduced expression of pro-inflammatory and pro-apoptotic genes in Makona infection compared to Ecran. These altered responses may reflect the differences in disease course observed in humans and macaques^1^,^5^. The reason for these observed cellular changes remains unexplored and may be due to functional differences in viral proteins. The small number of significant changes does not adequately demonstrate that the hypothesised variances in virus-host interactions are present. Interrogating the influence of transcription factors regulating these significant differentially expressed genes may reveal a dichotomy in regulation, or functional activity of the pathways highlighted in this study. Together this data indicated that the regulation of innate immune pathways appeared different in cells infected with the Makona variant compared with the Ecran variant and showed that these viral infections may have subtle phenotypic differences, and influence infection.

## Additional Information

**How to cite this article:** Bosworth, A. *et al*. A comparison of host gene expression signatures associated with infection *in vitro* by the Makona and Ecran (Mayinga) variants of Ebola virus. *Sci. Rep.* **7**, 43144; doi: 10.1038/srep43144 (2017).

**Publisher's note:** Springer Nature remains neutral with regard to jurisdictional claims in published maps and institutional affiliations.

## Supplementary Material

Supplementary Dataset 1

Supplemtary Data File

## Figures and Tables

**Table 1:** Mapped reads and mRNA counts per million.

	EBOV- Ecran			EBOV- Makona		
Sample	24 h p.i.	48 h p.i.	72 h p.i.	24 h p.i.	48 h p.i.	72 h p.i.
Trimmed reads	77,610,3339	77,211,979	67,874,000	102,746,478	57,588,535	66,319,784
						
Total Genes with mapped reads	26621	27146	26395	27895	25825	26541
Differentially Expressed Genes	11557	12865	13021	10456	12328	13239
Not Differentially Expressed Genes	15064	14281	13374	17439	13497	13302
Genes which increased in abundance	6093	7119	7319	5658	6674	7506
Genes which decreased in abundance	5464	5746	5702	4798	5654	5733

Table showing total trimmed reads acquired in each sample data set analysed over the 72 h experiment. The lower table indicates total genes in the transcriptomic analysis with mapped reads, divided further into those that were differentially expressed, and those that were not. This is then further divided into the number of genes with significant increase/decrease in abundance.

**Table 2:** Top 10 canonical pathways.

Top 10 Canonical Pathways in Makona Infection	
Pathway	Log- p-value
EIF2 Signalling	17.897
Mitochondrial Dysfunction	13.944
Oxidative Phosphorylation	12.862
Regulation of eIF4 and p7056k Signalling	10.436
mTOR Signalling	9.034
Oestrogen Receptor Signalling	6.547
CREB Signalling in Neurons	5.792
Superpathway of Cholesterol Biosynthesis	5.755
Protein Ubiquitination Pathway	5.213
Protein Kinase A Signalling	5.172

Table showing the top 10 canonical pathways to which all genes with a p-value of less than 0.001 during Makona infection were aligned in Ingenuity Pathway Analysis. Log p-value is shown as an indication of confidence in the functional grouping. Genes aligned to each pathway are functionally related and are connected either indirectly or directly to other components of the pathway.
