# Supplementary material for: A comparison of host gene expression signatures associated with infection in vitro by the Makona and Ecran (Mayinga) variants of Ebola virus
Source: Sci Rep. 2017 Feb 27;7:43144. doi: 10.1038/srep43144 (PMC5327407; doi:10.1038/srep43144)
Supplement: Supplemtary Data File [file srep43144-s2.pdf]

A comparison of host gene expression signatures associated with infection in vitro by the Makona and Ecran (Mayinga) variants of Ebola virus.

Mr. Andrew Bosworth , Dr. Stuart Dowall , Dr. Isabel Garcia-Dorival , Miss Natasha Y. Rickett , Dr. Christine B. Bruce , Dr. David A. Matthews , Dr. Yongxiang Fang , Dr. Waleed Aljabr , Dr. John Kenny , Ms. Charlotte Nelson , Dr. Thomas R. Laws , Prof. E. Diane Williamson , Prof. James P. Stewart , Prof. Miles Carroll , Prof. Roger Hewson, Prof. Julian A. Hiscox

Supplementary figures

Supplementary Figure 1: **Calculating significance of gene expressions**: (A) A dispersion plot generated with EdgeR using estimated dispersion of data in mock infected samples and then applying these to all datasets. The x-axis displays log Counts Per Million (CPM) and indicates the number of reads which map to each gene in the dataset, and the y-axis represents the level of data variation. This illustrates that genes with lower read counts display higher dispersion, and needs to be taken into account when calculating false-discovery rates. (B) Using the estimated dispersion false discovery rates were calculated and used to correct p-values for genes. Shown here are smear plots showing all genes with mapped reads in each dataset. Red dots indicate those which are calculated to be significantly changed based on dispersion data, and black dots indicate those which are not significant. These plots display Makona or Ecran compared with mock at 24h, 48h, and 72h time points. (C) Smear plots are shown for comparisons made directly between Makona and Ecran at 24h, 48h and 72h.

Supplementary Figure 2: **Global changes in canonical pathways**: This figure displays all canonical pathways to which genes aligned comparing either Makona or Ecran with mock infection at 24h, 48h and 72h. Red indicates an increase in pathway activation (z-score) and blue indicates a decrease in activation. White indicates no z-score has been assigned. It is clear that most pathways are showing increased z-score.

Supplementary Figure 3: **Expanded network of significant genes:** This figure displays all significant genes regardless of whether they are directly or indirectly connected to other genes, forming a network. This shows a wider view of the genes which are most different between Makona and Ecran compared with mock infection. Solid lines indicate direct interaction and dotted lines represent indirect interaction.

Supplementary Dataset File: **List of genes to which reads were mapped:** In addition to this manuscript an additional file is provided containing lists of genes to which reads were mapped for EBOV-Makona, EBOV –Ecran infections in A549 cells. Gene counts and fold change calculated using EdgeR in the R environment and arranged into lists by time post infection and either EBOV-Makona or EBOV-Ecran. P-values and FDR are also shown for each gene.

Supplementary Figure 1

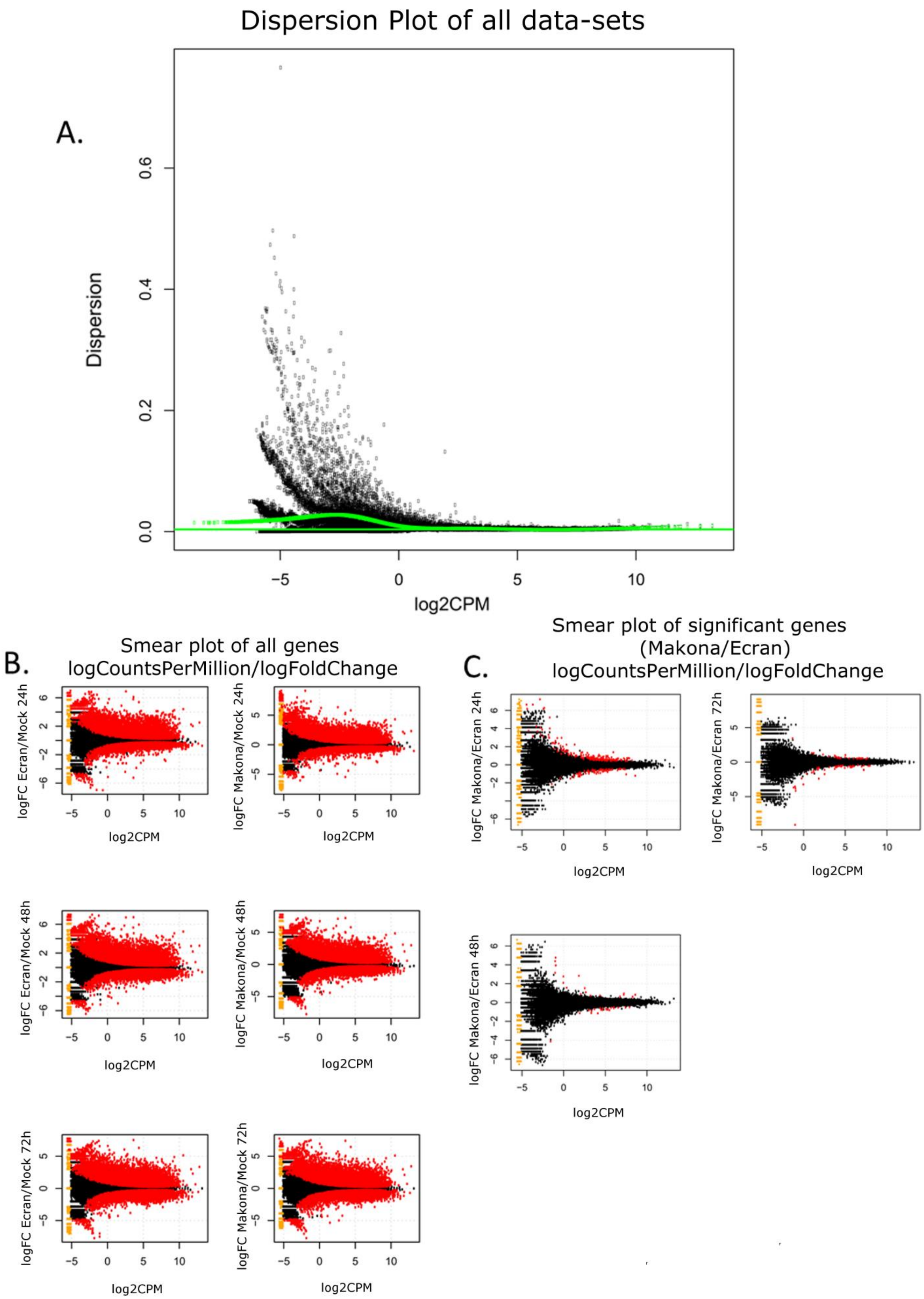

Supplementary Figure 2

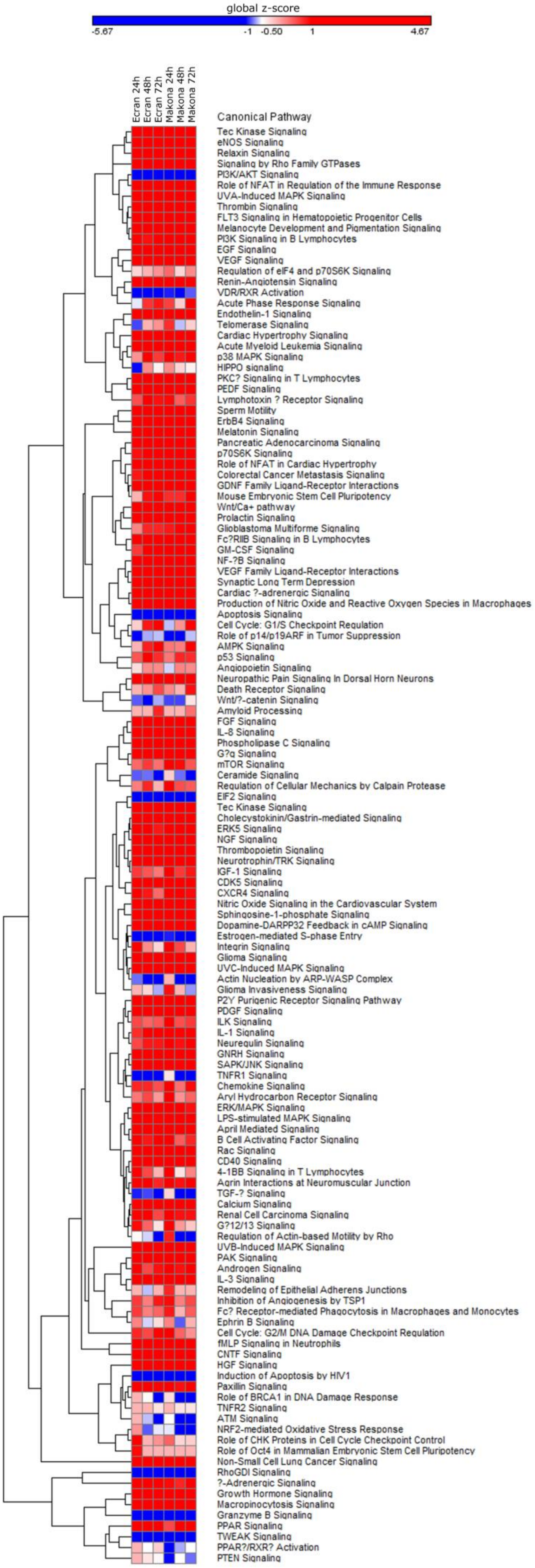

Supplementary Figure 3

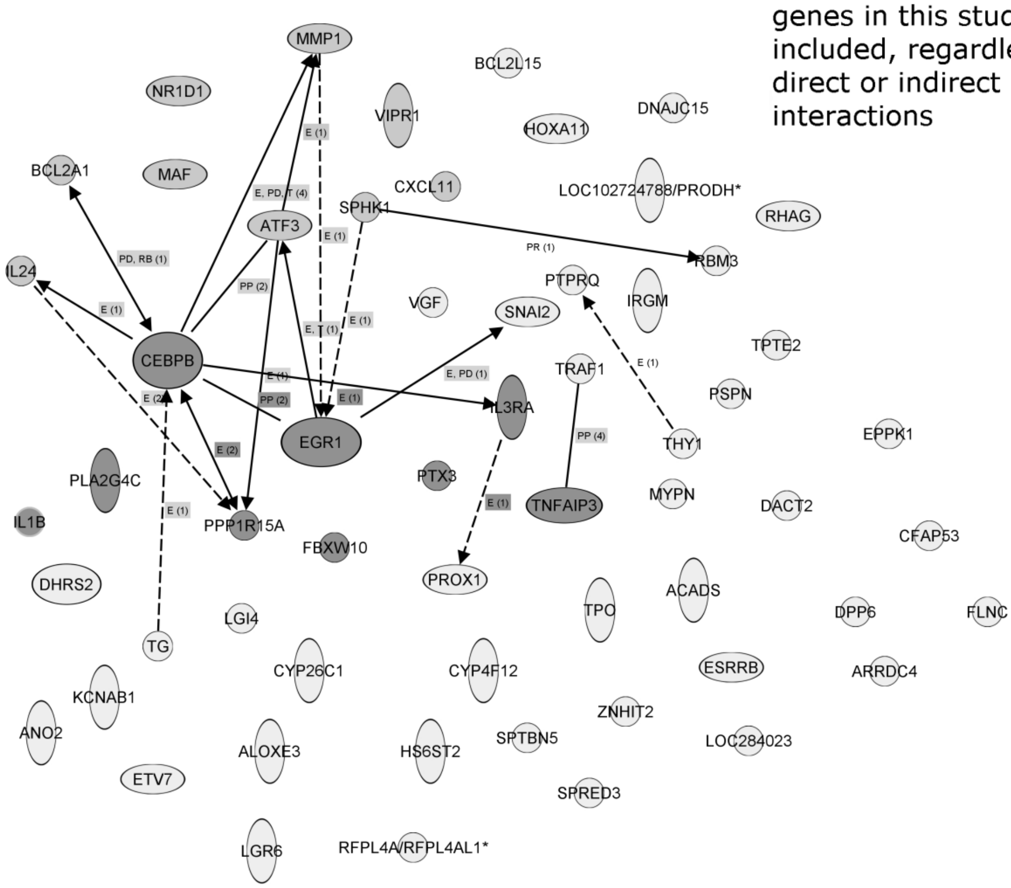

Expanded View

The most significant genes in this study are included, regardless of direct or indirect interactions
